# Data on the effects of imidazo[1,2-a]benzimidazole and pyrimido[1,2-a]benzimidazole compounds on intraocular pressure of ocular normotensive rats

**DOI:** 10.1016/j.dib.2018.03.019

**Published:** 2018-03-08

**Authors:** Adrian Julian Marcus, Igor Iezhitsa, Renu Agarwal, Pavel Vassiliev, Alexander Spasov, Olga Zhukovskaya, Vera Anisimova, Nafeeza Mohd Ismail

**Affiliations:** a Centre for Neuroscience Research, Faculty of Medicine, Universiti Teknologi MARA, Malaysia; b Volgograd State Medical University, Research Institute of Pharmacology, Volgograd, Russia; c Southern Federal University, Research Institute of Physical and Organic Chemistry, Rostov-on-Don, Russia

**Keywords:** Drug screening, Intraocular pressure, Intraocular pressure lowering activity, Imidazo[1,2-a]benzimidazoles, Pyrimido[1,2-a]benzimidazoles, Ocular normotensive rats, Rebound tonometry

## Abstract

This data is to document the intraocular pressure (IOP) lowering activity of imidazo[1,2-a]benzimidazole and pyrimido[1,2-a]benzimidazole compounds in ocular normotensive rats. Effects of single drop application of imidazo[1,2-a]benzimidazole and pyrimido[1,2-a]benzimidazole compounds on IOP in ocular normotensive rats are presented at 3 different concentrations (0.1%, 0.2% and 0.4%). Time course of changes in IOP is presented over 6 h period post-instillation. The IOP lowering activities of test compounds were determined by assessing maximum decrease in IOP from baseline and corresponding control, duration of IOP lowering and area under curve (AUC) of time versus response curve. Data shown here may serve as benchmarks for other researchers studying IOP-lowering effect of imidazo[1,2-a]benzimidazole and pyrimido[1,2-a]benzimidazole compounds and would be useful in determining therapeutic potential of these test compounds as IOP lowering agents.

**Specifications Table**

**Table:** 

Subject area	Medicine
More specific subject area	Ocular Pharmacology, Drug Screening, Drug Discovery
Type of data	Word 2016 text file
How data was acquired	This study was done in ocular normotensive rats and rebound tonometry (Tonolab, Icare Finland) was used to estimate intraocular pressure (IOP).
Data format	Analyzed
Experimental factors	This data is supplementary to [1, 2]. A total of 27 new compounds were synthesized as described previously [3], [4], [5], [6], [7], [8], [9], [10], [11] and tested for IOP lowering effect in rats. These compounds included twenty 9H-imidazo[1,2-a]benzimidazoles, four 10H-pyrimido[1,2-a]benzimidazoles, two 1H-pyrimido[1,2-a]benzimidazoles and one 1H-imidazo[1,2-a]benzimidazole.
Experimental features	All compounds were topically applied as a single drop, unilaterally, at 3 different concentrations (0.1%, 0.2% and 0.4%). The contralateral eye was instilled with vehicle and served as control. The IOP reduction was measured up to 6 h.
Data source location	Universiti Teknologi MARA, Faculty of Medicine, Sungai Buloh Campus, Jalan Hospital, 47000 Sungai Buloh, Selangor Darul Ehsan, Malaysia
Data accessibility	Data is with this article.
Related research article	This data is supplementary to [1, 2]

**Value of the Data**

• This data documented the IOP lowering activity for imidazo[1,2-a]benzimidazole and pyrimido[1,2-a]benzimidazole compounds in ocular normotensive rats.
• Effects of imidazo[1,2-a]benzimidazole and pyrimido[1,2-a]benzimidazole compounds on IOP in ocular normotensive rats are presented at 3 different concentrations (0.1%, 0.2% and 0.4%).
• Time course of changes in IOP are presented over 6 h post-instillation. The measurements were done at 6 time points (0, 0.5, 1, 1.5, 2, 3, 4, 5, 6 h). The IOP lowering activities of compounds were determined by assessing maximum decrease in IOP from baseline and corresponding control, duration of IOP lowering and area under curve (AUC) of time versus response curve.
• Data shown here may serve as benchmarks for other researchers studying IOP-lowering effect of imidazo[1,2-a]benzimidazole and pyrimido[1,2-a]benzimidazole compounds.
• This data would be useful in determining therapeutic potential of these test compounds as IOP lowering agents.

## 1 Data

Benzimidazoles are heterocyclic aromatic organic compound having structural analogy to nucleotides found in human body and hence are an important pharmacophore in medicinal chemistry [1], [2], [3], [4], [5], [6], [7], [8], [9], [10], [11], [12], [13], [14], [15]. They are known to exhibit Rho-kinase (ROCK) inhibitory activity and thus could be considered as novel potential class of anti-glaucoma therapeutics [14], [15], [16]. Data in this article is to present the effects of benzimidazole-based compounds on IOP of ocular normotensive rats and is supplementary to [1, 2]. These compounds included twenty 9H-imidazo[1,2-a]benzimidazoles, four 10H-pyrimido[1,2-a]benzimidazoles, two 1H-pyrimido[1,2-a]benzimidazoles and one 1H-imidazo[1,2-a]benzimidazole [1], [3], [4], [5], [6], [7], [8], [9], [10], [11].

### 1.1 9H-Imidazo[1,2-a]benzimidazole compounds

#### 1.1.1 RU 185

**Fig. 1:**
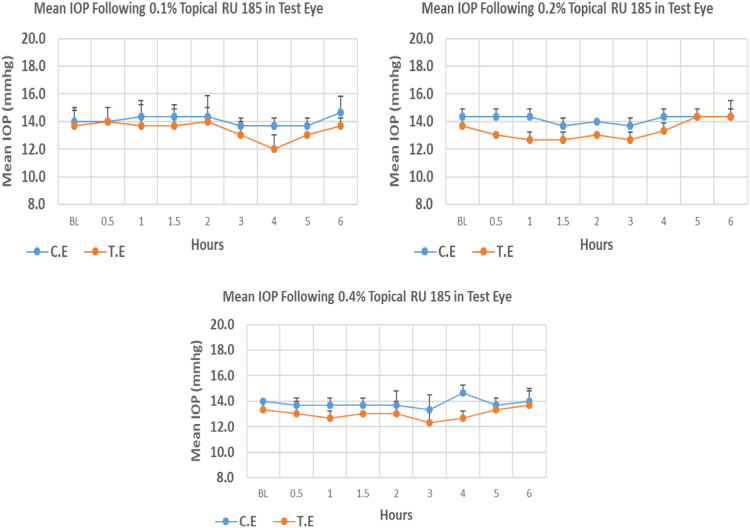
Effects of RU 185 on intraocular pressure of ocular normotensive rats in three different concentrations. TE – tested eye, CE – control eye, IOP - intraocular pressure.

RU 185 is a N9-imidazobenzimidazole derivative with molecular weight 526.2. At the onset of experiment there were no significant differences between test and control eyes. The 0.1% concentration of RU 185 caused higher reduction (12.22%) in IOP when compared to 0.2% and 0.4% concentrations with 7.32% and 7.50% reduction, respectively. However, none of these concentrations of RU 185 showed significant difference from baseline. Overall IOP lowering activities were represented by AUC of time versus response curve. An increase in AUC value was observed with increasing concentration (Fig. 1, Table 1).

**Table 1:** Effects of RU 185 on intraocular pressure of ocular normotensive rats in three different concentrations.

**RU 185 concentration %**	**Onset (h)**	**Max.** x̅ **%IOP Reduction BL vs TE**	***P*-value BL vs TE**	**Max.** x̅ **%IOP Reduction BL vs CE**	***P*-value BL vs CE**	**AUC**
**0.1**	–	−12.22	0.132	−2.38	0.643	4.50
**0.2**	–	−7.32	0.101	−4.65	0.230	4.90
**0.4**	–	−7.50	0.251	−4.76	0.374	5.30

BL – base line, TE – tested eye, CE – control eye, IOP - intraocular pressure, AUC – area under curve of time versus response curve.

#### 1.1.2 RU 238

**Fig. 2:**
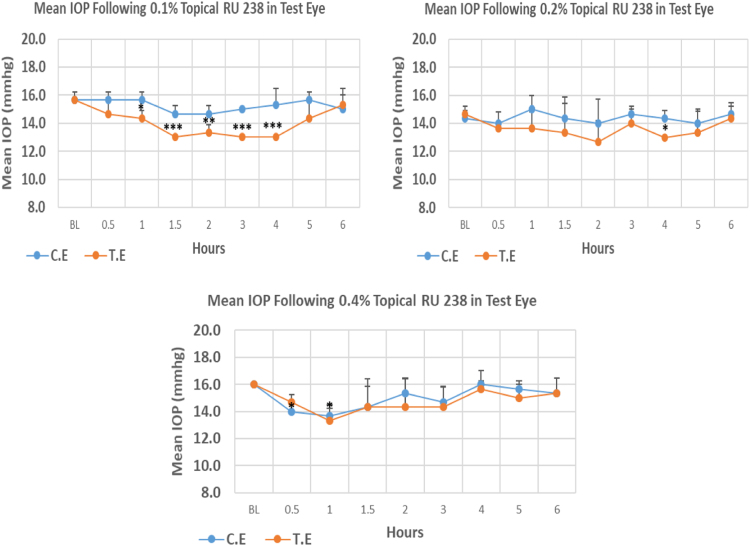
Effects of RU 238 on intraocular pressure of ocular normotensive rats in three different concentrations. *P-value ≤ 0.05, **P-value ≤0.01, and ***P-value ≤0.001; TE – tested eye, CE – control eye, IOP - intraocular pressure.

RU 238 is a N9-imidazobenzimidazole derivative with molecular weight 431.4. The onset of IOP reduction compared to baseline with 0.1%, 0.2% and 0.4% was at 1, 0.5 and 4 h, post-instillation, respectively. The peak IOP reduction with a mean value of 17.04% at 0.1% concentration was higher when compared to 0.2% and 0.4% concentrations with mean values of 13.63% and 16.69%, respectively. The mean IOP at the time of maximum lowering with 0.1% and 0.4% concentration was significantly lower than baseline but the same was not observed with 0.2% concentration. Overall IOP lowering activities were represented by AUC of time versus response curve. With an increase in concentration, there was a trend towards decrease in AUC value (Fig. 2, Table 2).

**Table 2:** Effects of RU 238 on intraocular pressure of ocular normotensive rats.

**RU 238 concentration %**	**Onset (h)**	**Max.** x̅ **%IOP Reduction BL vs TE**	***P*-value BL vs TE**	**Max.** x̅ **%IOP Reduction BL vs CE**	***P*-value BL vs CE**	**AUC**
**0.1**	1.00	−17.04	0.001	−6.38	0.101	8.10
**0.2**	4.00	−13.63	0.101	−2.32	0.374	6.10
**0.4**	0.50	−16.69	0.016	−14.58	0.002	1.80

BL – base line, TE – tested eye, CE – control eye, IOP - intraocular pressure, AUC – area under curve of time versus response curve.

#### 1.1.3 RU 239

**Fig. 3:**
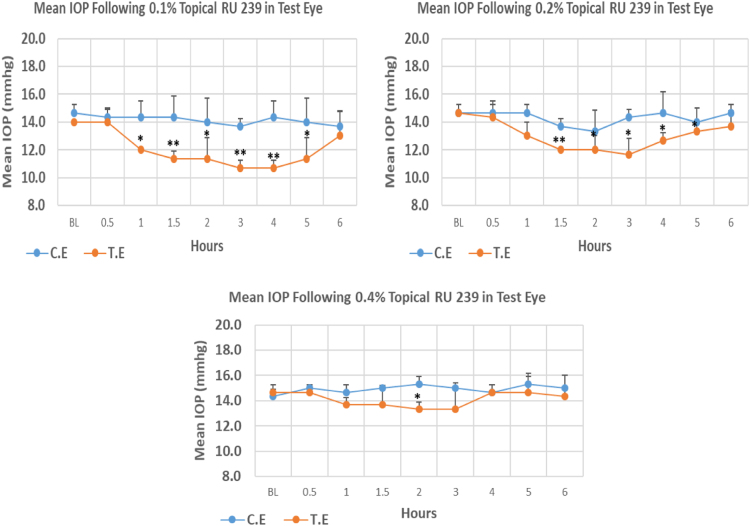
Effects of RU 239 on intraocular pressure of ocular normotensive rats in three different concentrations. *P-value ≤0.05, **P-value ≤0.01, and ***P-value ≤0.001. TE – tested eye, CE – control eye, IOP - intraocular pressure.

RU 239 is a N9-imidazobenzimidazole derivative with molecular weight 433.3. The onset of IOP lowering with 0.1%, 0.2% and 0.4% concentrations was 1, 1.5 and 2 h post-treatment, respectively. In terms of maximum IOP reduction, 0.1% caused 23.79% IOP reduction from baseline which was higher when compared to 0.2% and 0.4% concentrations with 20.45% and 9.13% reduction respectively. At all three-concentrations mean IOP was significantly lower than baseline. Overall IOP lowering activities were represented by AUC value. Interestingly, with increase in concentration, there was a trend towards decrease in AUC value (Fig. 3, Table 3).

**Table 3:** Effects of RU 239 on intraocular pressure of ocular normotensive rats.

**RU 239 concentration %**	**Onset (h)**	**Max.** x̅ **%IOP Reduction BL vs TE**	***P*-value BL vs TE**	**Max.** x̅ **%IOP Reduction BL vs CE**	***P*-value BL vs CE**	**AUC**
**0.1**	1.00	−23.79	0.001	−6.82	0.101	14.70
**0.2**	1.50	−20.45	0.016	−9.09	0.230	8.70
**0.4**	2.00	−9.13	0.047	+2.33	0.519	4.50

*Note*: BL – base line, TE – tested eye, CE – control eye, IOP - intraocular pressure, AUC – area under curve of time versus response curve.

#### 1.1.4 RU 243

**Fig. 4:**
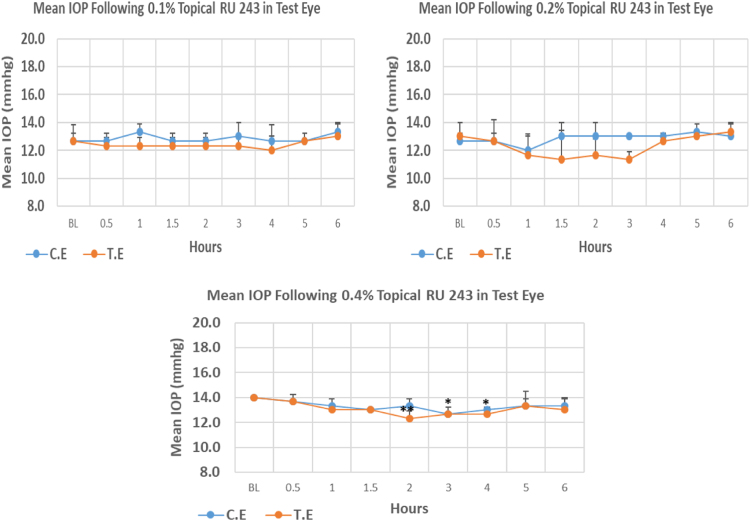
Effects of RU 243 on intraocular pressure of ocular normotensive rats in three different concentrations. *P-value ≤0.05, **P-value ≤0.01, and ***P-value ≤0.001. TE – tested eye, CE – control eye, IOP - intraocular pressure.

RU 243 is a N9-imidazobenzimidazole derivative with molecular weight 459.4. Its 0.1% and 0.2% concentrations did not cause significant IOP reduction compared to baseline. Whereas, 0.4% concentration caused onset of significant IOP reduction at 2.00 h post-instillation. Peak IOP reduction with 0.4% concentration compared to baseline amounted to 14.29%. Overall IOP lowering activities were represented by AUC values and increasing concentrations resulted in decreasing AUC values (Fig. 4, Table 4).

**Table 4:** Effects of RU 243 on intraocular pressure of ocular normotensive rats.

**RU 243 concentration %**	**Onset (h)**	**Max.** x̅ **%IOP Reduction BL vs TE**	***P*-value BL vs TE**	**Max.** x̅ **%IOP Reduction BL vs CE**	***P*-value BL vs CE**	**AUC**
**0.1**	–	−5.29	0.492	−0	–	10.29
**0.2**	–	−12.85	0.279	−5.27	0.374	5.35
**0.4**	2.00	−14.29	0.007	−9.52	0.016	2.75

BL – base line, TE – tested eye, CE – control eye, IOP - intraocular pressure, AUC – area under curve of time versus response curve.

#### 1.1.5 RU 244

**Fig. 5:**
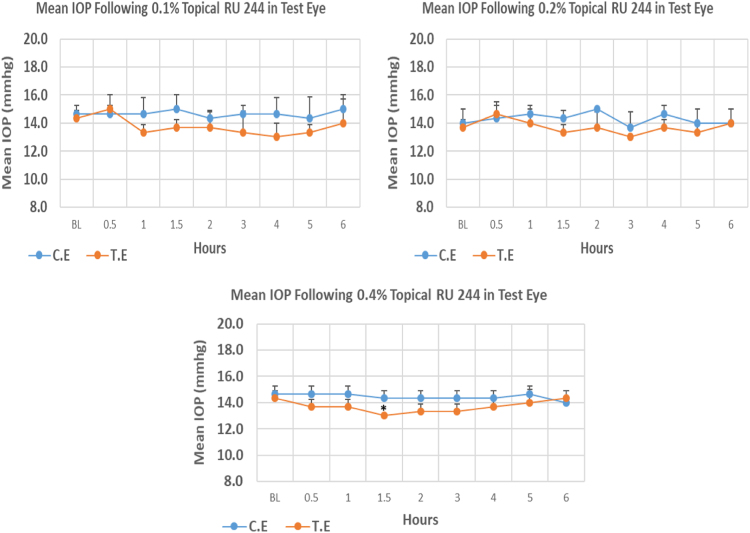
Effects of RU 244 on intraocular pressure of ocular normotensive rats in three different concentrations. *P-value ≤0.05, **P-value ≤0.01, and ***P-value ≤0.001. TE – tested eye, CE – control eye, IOP - intraocular pressure.

RU 244 is a N9-imidazobenzimidazole derivative with molecular weight 461.4. Its 0.1% and 0.2% concentrations did not cause significant IOP reduction compared to baseline. Whereas, 0.4% concentration caused onset of significant IOP reduction at 1.50 h post-instillation. The mean IOP at the time of maximum lowering with 0.4% concentration was significantly lower than baseline but the same was not observed with 0.1%, and 0.2% concentrations. Overall IOP lowering activities were represented by AUC value where increasing concentrations resulted in decreasing AUC values (Fig. 5, Table 5).

**Table 5:** Effects of RU 244 on intraocular pressure of ocular normotensive rats.

**RU 244 concentration %**	**Onset (h)**	**Max.** x̅ **%IOP Reduction BL vs TE**	***P*-value BL vs TE**	**Max.** x̅ **%IOP Reduction BL vs CE**	***P*-value BL vs CE**	**AUC**
**0.1**	–	−9.28	0.116	−2.28	0.519	6.60
**0.2**	–	−4.90	0.116	−4.88	0.725	4.30
**0.4**	1.5	−9.28	0.016	−9.30	0.519	4.70

BL – base line, TE – tested eye, CE – control eye, IOP - intraocular pressure, AUC – area under curve of time versus response curve.

#### 1.1.6 RU 247

**Fig. 6:**
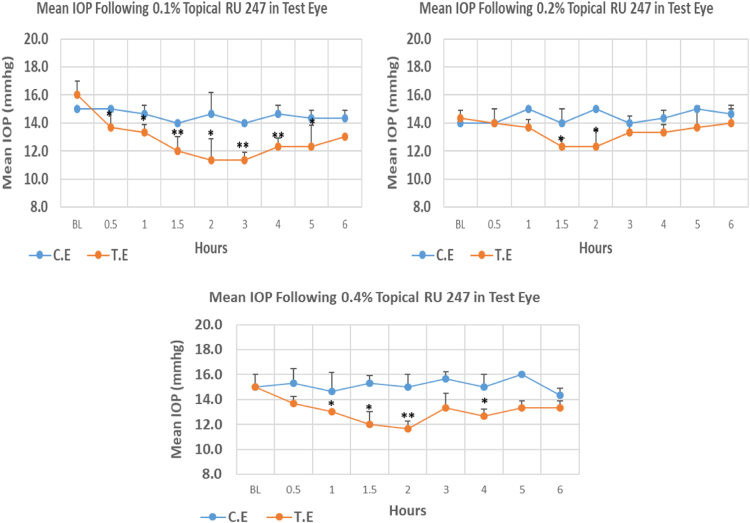
Effects of RU 247 on intraocular pressure of ocular normotensive rats in three different concentrations. *P-value≤0.05, **P-value≤0.01, and ***P-value≤0.001. TE – tested eye, CE – control eye, IOP - intraocular pressure.

RU 247 is a N9-imidazobenzimidazole derivative with molecular weight 431.4. The onset of IOP lowering with 0.1%, 0.2% and 0.4% concentrations was 0.5, 1.5 and 1.0 h post-treatment, respectively. The 0.1% concentration of RU 247 caused higher reduction (29.19%) in IOP when compared to 0.2% and 0.4% concentrations with 13.96% and 22.20% reduction, respectively. All three concentrations showed significant differences in maximum IOP reduction. Overall IOP lowering activities were represented by AUC values. Interestingly, the IOP reduction with this compound was concentration-independent (Fig. 6, Table 6).

**Table 6:** Effects of RU 247 on intraocular pressure of ocular normotensive rats.

**RU 247 concentration %**	**Onset (h)**	**Max.** x̅ **%IOP Reduction BL vs TE**	***P*-value BL vs TE**	**Max.** x̅ **%IOP Reduction BL vs CE**	***P*-value BL vs CE**	**AUC**
**0.1**	0.50	−29.19	0.011	−8.89	0.016	12.30
**0.2**	1.50	−13.96	0.013	−0	0	7.50
**0.4**	1.00	−22.20	0.007	−4.45	0.374	16.10

BL – base line, TE – tested eye, CE – control eye, IOP - intraocular pressure, AUC – area under curve of time versus response curve.

#### 1.1.7 RU 284

**Fig. 7:**
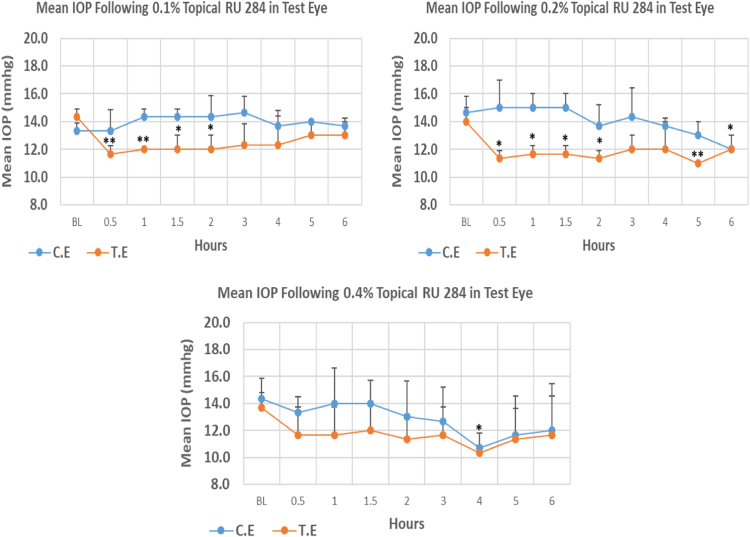
Effects of RU 284 on intraocular pressure of ocular normotensive rats in three different concentrations. *P-value≤0.05, **P-value≤0.01, and ***P-value≤0.001. TE – tested eye, CE – control eye, IOP - intraocular pressure.

RU 284 is a N9-imidazobenzimidazole derivative with molecular weight 419.3. The onset of IOP lowering with both 0.1%, and 0.2% concentration was 0.5 h post-treatment. Whereas at higher concentration, 0.4%, the onset of IOP lowering was 4.0 h post-treatment. The mean IOP at the time of maximum lowering for all three concentrations was significantly lower than baseline. Overall IOP lowering activities were represented by AUC values. Both 0.1 and 0.2% concentrations demonstrated higher AUC when compared to 0.4% concentration (Fig. 7, Table 7).

**Table 7:** Effects of RU 284 on intraocular pressure of ocular normotensive rats.

**RU 284 concentration %**	**Onset (h)**	**Max.** x̅ **%IOP Reduction BL vs TE**	***P*-value BL vs TE**	**Max.** x̅ **%IOP Reduction BL vs CE**	***P*-value BL vs CE**	**AUC**
**0.1**	0.50	−18.56	0.005	−0	–	11.83
**0.2**	0.50	−21.43	0.007	−18.18	0.039	13.75
**0.4**	4.00	−24.43	0.011	−25.58	0.029	6.25

BL – base line, TE – tested eye, CE – control eye, IOP - intraocular pressure, AUC – area under curve of time versus response curve.

#### 1.1.8 RU 412

**Fig. 8:**
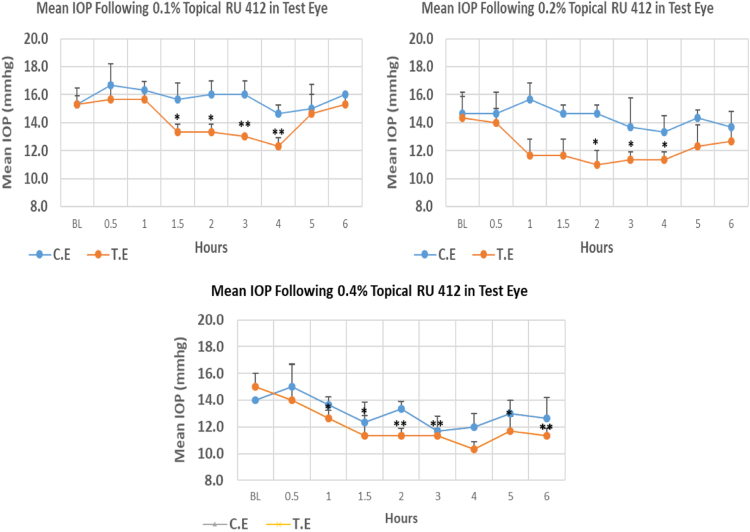
Effects of RU 412 on intraocular pressure of ocular normotensive rats in three different concentrations. *P-value ≤0.05, **P-value ≤0.01, and ***P-value ≤0.001. TE – tested eye, CE – control eye, IOP - intraocular pressure.

RU 412 is a N9-imidazobenzimidazole derivative with molecular weight 419.3. The onset of IOP reduction compared to baseline with 0.1%, 0.2% and 0.4% was 1.5, 2.0, and 1.0 h, post-instillation, respectively. The maximum IOP reduction from baseline for 0.1%, 0.2%, and 0.4% concentrations was 19.57%, 23.24%, and 31.13% respectively. All three concentrations showed significant differences in maximum IOP reduction from baseline. Overall IOP lowering activities were represented by AUC values. Both 0.1 and 0.2% concentrations demonstrated higher AUC when compared to 0.4% concentration (Fig. 8, Table 8).

**Table 8:** Effects of RU 412 on intraocular pressure of ocular normotensive rats.

**RU 412 concentration %**	**Onset (h)**	**Max.** x̅ **%IOP Reduction BL vs TE**	***P*-value BL vs TE**	**Max.** x̅ **%IOP Reduction BL vs CE**	***P*-value BL vs CE**	**AUC**
**0.1**	1.50	−19.57	0.003	−4.34	0.422	12.20
**0.2**	2.00	−23.24	0.034	−9.10	0.294	13.50
**0.4**	1.00	−31.13	0.002	−16.66	0.025	6.80

BL – base line, TE – tested eye, CE – control eye, IOP - intraocular pressure, AUC – area under curve of time versus response curve.

#### 1.1.9 RU 437

**Fig. 9:**
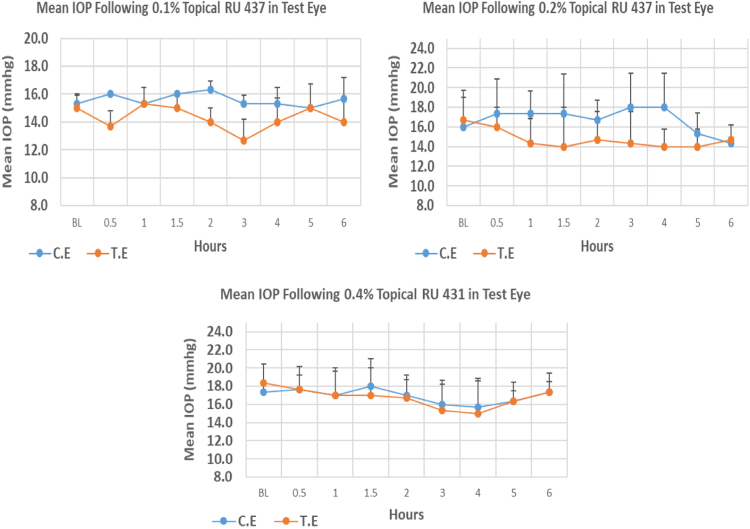
Effects of RU 437 on intraocular pressure of ocular normotensive rats in three different concentrations. TE – tested eye, CE – control eye, IOP - intraocular pressure.

RU 437 is a N9-imidazobenzimidazole derivative with molecular weight 465.4. The maximum IOP reduction from baseline for 0.1%, 0.2%, and 0.4% was 15.53%, 16.02%, and 18.17% respectively. However, none of the three concentrations showed significant differences at the time of maximum IOP reduction from baseline. Overall IOP lowering activities were represented by AUC values. Both 0.1% and 0.2% concentrations demonstrated higher AUC when compared to 0.4% concentration (Fig. 9, Table 9).

**Table 9:** Effects of RU 437 on intraocular pressure of ocular normotensive rats.

**RU 437 concentration %**	**Onset (h)**	**Max.** x̅ **%IOP Reduction BL vs TE**	***P*-value BL vs TE**	**Max.** x̅ **%IOP Reduction BL vs CE**	***P*-value BL vs CE**	**AUC**
**0.1**	–	–15.53	0.091	–2.17	0.768	8.30
**0.2**	–	–16.02	0.411	–10.42	0.398	14.00
**0.4**	–	–18.17	0.238	–9.61	0.446	1.80

BL – base line, TE – tested eye, CE – control eye, IOP - intraocular pressure, AUC – area under curve of time versus response curve.

#### 1.1.10 RU 438

**Fig. 10:**
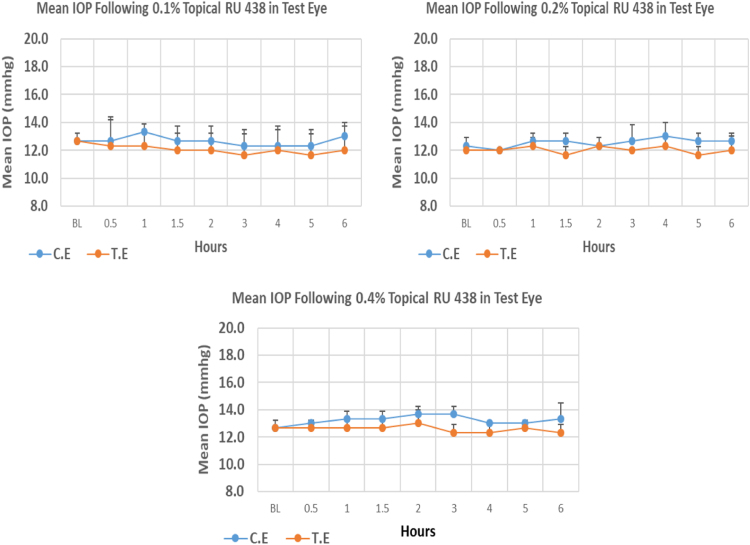
Effects of RU 438 on intraocular pressure of ocular normotensive rats in three different concentrations. TE – tested eye, CE – control eye, IOP - intraocular pressure.

RU 438 is a N9-imidazobenzimidazole derivative with molecular weight 626.5. The maximum IOP reduction from baseline for 0.1%, 0.2%, and 0.4% concentration was 7.89%, 2.75%, and 2.68% respectively. However, none of the three concentrations showed significant differences at the time of maximum IOP reduction. Overall IOP lowering activities were represented by AUC values. Both 0.1 and 0.2% concentrations demonstrated higher AUC when compared to 0.4% concentration (Fig. 10, Table 10).

**Table 10:** Effects of RU 438 on intraocular pressure of ocular normotensive rats.

**RU 438 concentration %**	**Onset (h)**	**Max.** x̅ **%IOP Reduction BL vs TE**	***P*-value BL vs TE**	**Max.** x̅ **%IOP Reduction BL vs CE**	***P*-value BL vs CE**	**AUC**
**0.1**	–	−7.89	0.349	−2.64	0.678	8.30
**0.2**	–	−2.75	0.374	−2.70	0.374	14.00
**0.4**	–	−2.68	0.519	−0	–	1.80

BL – base line, TE – tested eye, CE – control eye, IOP - intraocular pressure, AUC – area under curve of time versus response curve.

#### 1.1.11 RU 441

**Fig. 11:**
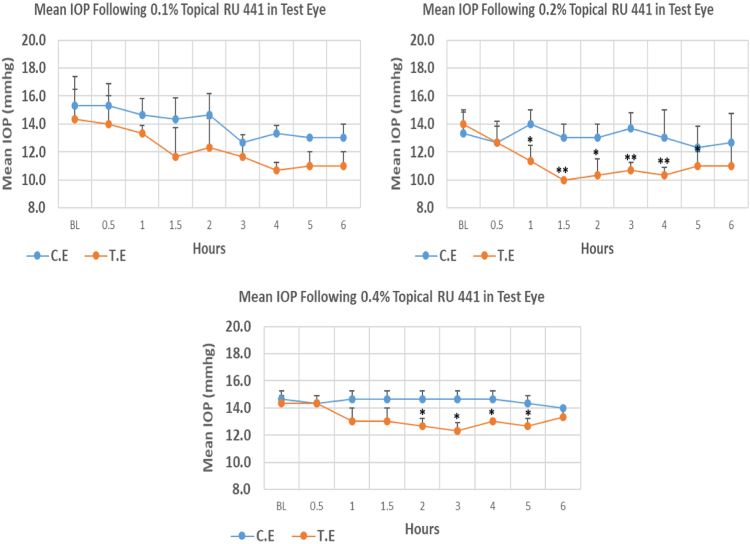
Effects of RU 441 on intraocular pressure of ocular normotensive rats in three different concentrations. *P-value≤0.05, **P-value≤0.01, and ***P-value≤0.001. TE – tested eye, CE – control eye, IOP - intraocular pressure.

RU 441 is a N9-imidazobenzimidazole derivative with molecular weight 443.3. Its 0.1% concentrations did not cause significant IOP reduction compared to baseline at any time point. Whereas, 0.2, and 0.4% concentrations caused onset of significant IOP reduction at 1.00, and 2.00 h post-instillation respectively. The mean IOP at the time of peak IOP reduction with 0.2% and 0.4% was significantly lower than baseline. Overall IOP lowering activities were represented by AUC values. Both 0.1% and 0.2% concentrations demonstrated higher AUC when compared to 0.4% concentration (Fig. 11, Table 11).

**Table 11:** Effects of RU 441 on intraocular pressure of ocular normotensive rats.

**RU 441 concentration %**	**Onset (h)**	**Max.** x̅ **%IOP Reduction BL vs TE**	***P*-value BL vs TE**	**Max.** x̅ **%IOP Reduction BL vs CE**	***P*-value BL vs CE**	**AUC**
**0.1**	–	−25.54	0.111	−17.39	0.023	11.53
**0.2**	1.00	−28.57	0.002	−7.50	0.468	12.53
**0.4**	2.00	−13.95	0.013	−4.55	0.116	8.90

BL – base line, TE – tested eye, CE – control eye, IOP - intraocular pressure, AUC – area under curve of time versus response curve.

#### 1.1.12 RU 477

**Fig. 12:**
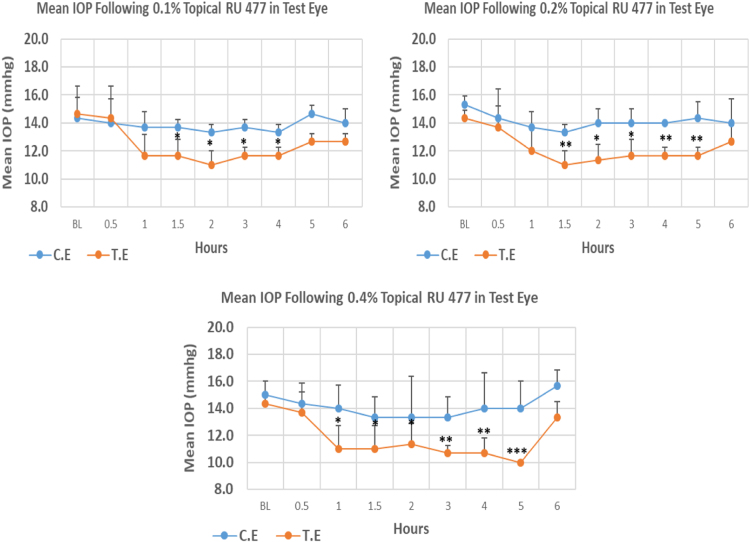
Effects of RU 477 on intraocular pressure of ocular normotensive rats in three different concentrations. *P-value≤0.05, **P-value≤0.01, and ***P-value≤0.001. TE – tested eye, CE – control eye, IOP - intraocular pressure.

RU 477 is a N9-imidazobenzimidazole derivative with molecular weight 524.2. The onset of IOP lowering with 0.1%, 0.2% and 0.4% concentrations was 1.5, 1.5 and 1.0 h post-treatment, respectively. In terms of maximum reduction, 0.1%, 0.2%, and 0.4% concentration demonstrated dose independent effects with 25.02%, 23.24%, and 30.22% IOP reduction from baseline, respectively. The mean IOP at the time of maximum lowering for all three concentrations was significantly lower than baseline. Overall IOP lowering activities were represented by AUC values. Increasing concentrations resulted in increasing AUC value (Fig. 12,Table 12).

**Table 12:** Effects of RU 477 on intraocular pressure of ocular normotensive rats.

**RU 477 concentration %**	**Onset (h)**	**Max.** x̅ **%IOP Reduction BL vs TE**	***P*-value BL vs TE**	**Max.** x̅ **%IOP Reduction BL vs CE**	***P*-value BL vs CE**	**AUC**
**0.1**	1.5	−25.02	0.014	−6.98	0.507	8.80
**0.2**	1.5	−23.24	0.007	−13.04	0.013	12.40
**0.4**	1.0	−30.22	0.0002	−11.11	0.189	15.50

BL – base line, TE – tested eye, CE – control eye, IOP - intraocular pressure, AUC – area under curve of time versus response curve.

#### 1.1.13 RU 487

**Fig. 13:**
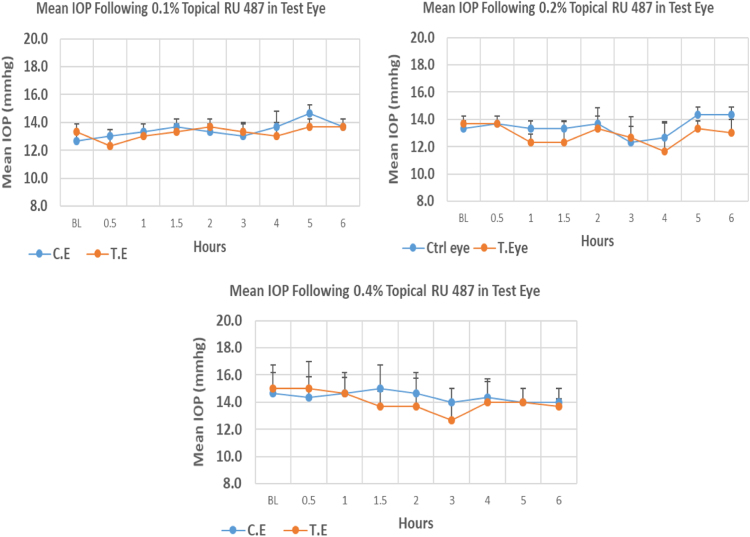
Effects of RU 487 on intraocular pressure of ocular normotensive rats in three different concentrations. TE – tested eye, CE – control eye, IOP - intraocular pressure.

RU 487 is a N9-imidazobenzimidazole derivative with molecular weight 504.2. The onset of IOP lowering with 0.1%, 0.2% and 0.4% concentrations was 0.5, 1.0 and 1.0 h post-treatment, respectively. The maximum IOP reduction from baseline for 0.1%, 0.2%, and 0.4% was 15.53%, 16.02%, and 18.17% respectively. However, none of the three concentrations showed significant differences from baseline at the time of maximum IOP reduction. Overall IOP lowering activities were represented by AUC values. Interestingly, this compound showed a concentration-independent effect on the AUC values (Fig. 13, Table 13).

**Table 13:** Effects of RU 487 on intraocular pressure of ocular normotensive rats.

**RU 487 concentration %**	**Onset (h)**	**Max.** x̅ **%IOP Reduction BL vs TE**	***P*-value BL vs TE**	**Max.** x̅ **%IOP Reduction BL vs CE**	***P*-value BL vs CE**	**AUC**
**0.1**	0.5	−7.50	0.251	+2.63	0.643	1.60
**0.2**	1.0	−14.63	0.184	−7.50	0.251	3.50
**0.4**	1.0	−15.53	0.124	−4.55	0.561	2.80

BL – base line, TE – tested eye, CE – control eye, IOP - intraocular pressure, AUC – area under curve of time versus response curve.

#### 1.1.14 RU 490

**Fig. 14:**
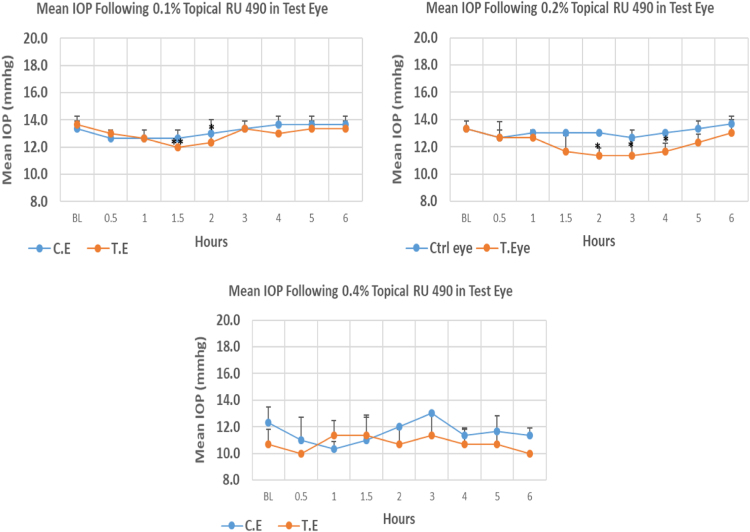
Effects of RU 490 on intraocular pressure of ocular normotensive rats in three different concentrations. TE – tested eye, CE – control eye, IOP - intraocular pressure.

RU 490 is a N9-imidazobenzimidazole derivative with molecular weight 477.4 The onset of IOP lowering with 0.1%, and 0.2% concentrations was 1.5, and 2.0 post-treatment, respectively. The maximum IOP reduction from baseline for 0.1%, 0.2%, and 0.4% was 12.20%, 15.00%, and 6.25%, respectively. Only 0.1% and 0.2% concentrations caused significant drop in mean IOP values at the time of maximum IOP reduction from baseline. Overall IOP lowering activities were represented by AUC values. An increase in AUC value was observed with increasing concentration (Fig. 14, Table 14).

**Table 14:** Effects of RU 490 on intraocular pressure of ocular normotensive rats.

**RU 490 concentration %**	**Onset (h)**	**Max.** x̅ **%IOP Reduction BL vs TE**	***P*-value BL vs TE**	**Max.** x̅ **%IOP Reduction BL vs CE**	***P*-value BL vs CE**	**AUC**
**0.1**	1.50	−12.20	0.007	−5.00	0.230	1.80
**0.2**	2.00	−15.00	0.013	−5.00	0.230	6.10
**0.4**	–	−6.25	0.374	−16.22	0.055	5.30

BL – base line, TE – tested eye, CE – control eye, IOP - intraocular pressure, AUC – area under curve of time versus response curve.

#### 1.1.15 RU 519

**Fig. 15:**
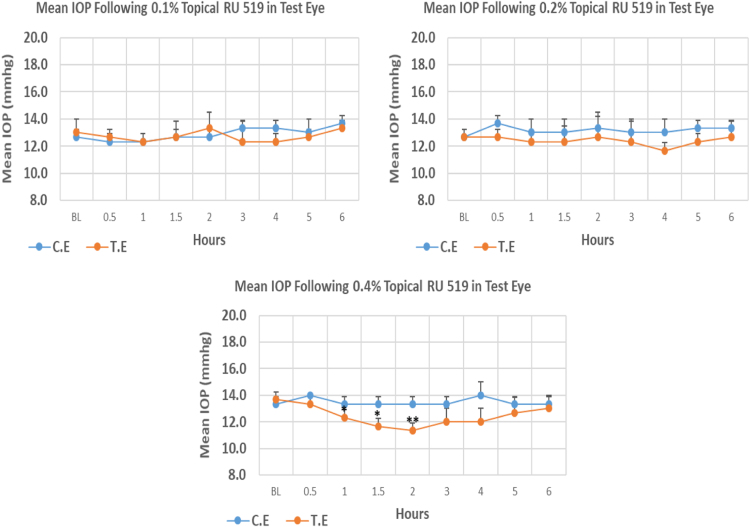
Effects of RU 519 on intraocular pressure of ocular normotensive rats in three different concentrations. *P-value≤0.05, **P-value≤0.01, and ***P-value≤0.001. TE – tested eye, CE – control eye, IOP - intraocular pressure.

RU 519 is a N9-imidazobenzimidazole derivative with molecular weight 632.8. Its 0.1% and 0.2% concentrations did not cause significant IOP reduction compared to baseline at any time point. Whereas, 0.4% concentration caused onset of significant IOP reduction at 1.00 h post-instillation. The maximum IOP reduction from baseline for 0.1%, 0.2%, and 0.4% was 5.15%, 7.89%, and 17.08%, respectively. Overall IOP lowering activities were represented by AUC values. An increase in AUC value was observed with increasing concentration (Fig. 15, Table 15).

**Table 15:** Effects of RU 519 on intraocular pressure of ocular normotensive rats.

**RU 519 concentration %**	**Onset (h)**	**Max.** x̅ **%IOP Reduction BL vs TE**	***P*-value BL vs TE**	**Max.** x̅ **%IOP Reduction BL vs CE**	***P*-value BL vs CE**	**AUC**
**0.1**	–	−5.15	0.374	+7.89	0.101	5.30
**0.2**	–	−7.89	0.101	+7.89	0.101	4.30
**0.4**	1.00	−17.08	0.008	+5.00	0.374	7.30

BL – base line, TE – tested eye, CE – control eye, IOP - intraocular pressure, AUC – area under curve of time versus response curve.

#### 1.1.16 RU 615

**Fig. 16:**
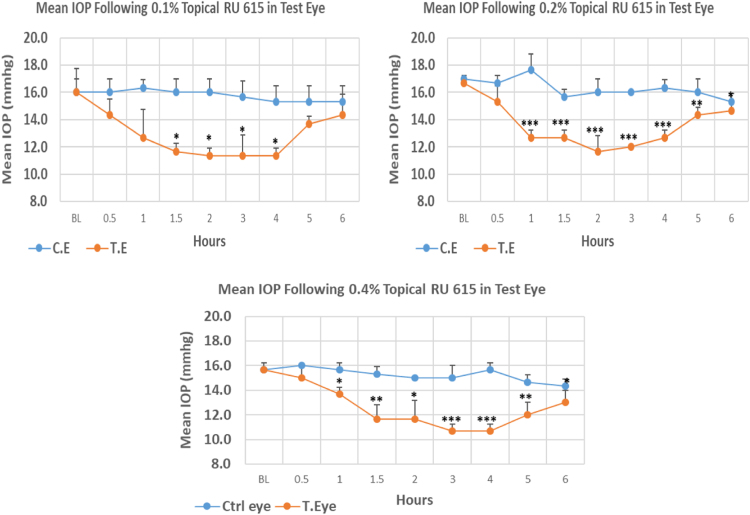
Effects of RU 615 on intraocular pressure of ocular normotensive rats in three different concentrations. *P-value≤0.05, **P-value≤0.01, and ***P-value≤0.001. TE – tested eye, CE – control eye, IOP – intraocular pressure.

RU 615 is a N9-imidazobenzimidazoles derivative with molecular weight 447.4. This derivative demonstrated early onset of significant IOP reduction with 0.5 h post-treatment for 0.1% concentration and 1.0 h for both 0.2% and 0.4% concentrations. The maximum IOP reduction from baseline for 0.1%, 0.2%, and 0.4% concentrations was 29.19%, 30.00%, and 31.19%, respectively. All three concentrations showed significant differences in mean IOP values from baseline at the time of maximum IOP reduction from baseline. Overall IOP lowering activities were represented by AUC values (Fig. 16, Table 16).

**Table 16:** Effects of RU 615 on intraocular pressure of ocular normotensive rats.

**RU 615 concentration %**	**Onset (h)**	**Max.** x̅ **%IOP Reduction BL vs TE**	***P*-value BL vs TE**	**Max.** x̅ **%IOP Reduction BL vs CE**	***P*-value BL vs CE**	**AUC**
**0.1**	1.5	−29.19	0.011	−4.17	0.492	18.80
**0.2**	1.0	−30.00	0.003	−9.81	0.007	17.20
**0.4**	1.0	−31.19	0.0004	−8.51	0.047	18.50

BL – base line, TE – tested eye, CE – control eye, IOP - intraocular pressure, AUC – area under curve of time versus response curve.

#### 1.1.17 RU 616

**Fig. 17:**
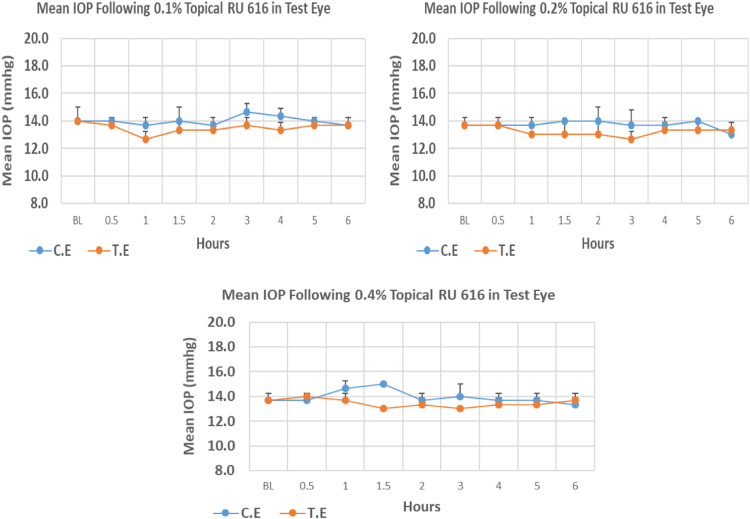
Effects of RU 616 on intraocular pressure of ocular normotensive rats in three different concentrations. TE – tested eye, CE – control eye, IOP – intraocular pressure.

RU 616 is a N9-imidazobenzimidazole derivative with molecular weight 449.3. None of the 0.1%, 0.2% and 0.4% concentrations showed significant IOP reduction from baseline at any time point over 6 h post-instillation (Fig. 17, Table 17).

**Table 17:** Effects of RU 616 on intraocular pressure of ocular normotensive rats.

**RU 616 concentration %**	**Onset (h)**	**Max.** x̅ **%IOP Reduction BL vs TE**	***P*-value BL vs TE**	**Max.** x̅ **%IOP Reduction BL vs CE**	***P*-value BL vs CE**	**AUC**
**0.1**	–	−9.5	0.116	−2.38	0.643	3.6
**0.2**	–	−7.32	0.101	−4.88	0.116	3.4
**0.4**	–	−4.9	0.116	−2.44	0.519	2.8

BL – base line, TE – tested eye, CE – control eye, IOP - intraocular pressure, AUC – area under curve of time versus response curve.

#### 1.1.18 RU 828

**Fig. 18:**
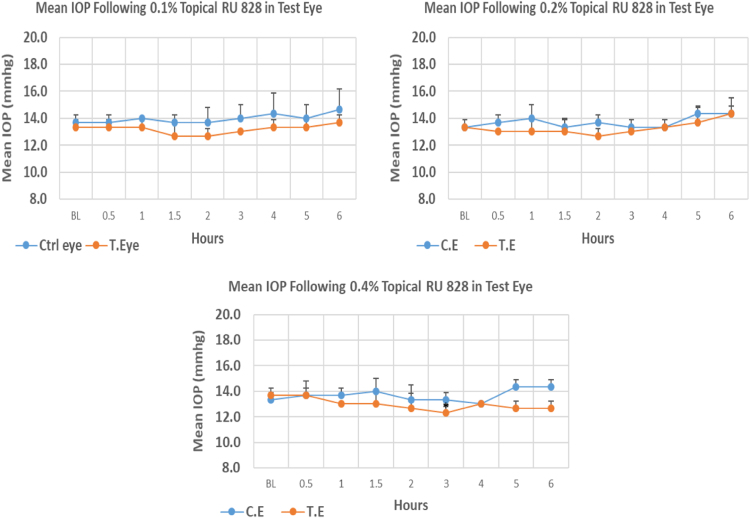
Effects of RU 828 on intraocular pressure of ocular normotensive rats in three different concentrations. *P-value≤0.05, **P-value≤0.01, and ***P-value≤0.001. TE – tested eye, CE – control eye, IOP – intraocular pressure.

RU 828 is a N9-imidazobenzimidazole derivative with molecular weight 329.2. The onset of IOP lowering with 0.4% concentrations was 3.0 h post-treatment. Whereas, 0.1%, and 0.2% concentrations showed no significant difference from baseline over the entire period of observation. Only 0.4% concentration showed significant IOP lowering compared to baseline at 3 h post-instillation (Fig. 18, Table 18).

**Table 18:** Effects of RU 828 on intraocular pressure of ocular normotensive rats.

**RU 828 concentration %**	**Onset (h)**	**Max.** x̅ **%IOP Reduction BL vs TE**	***P*-value BL vs TE**	**Max.** x̅ **%IOP Reduction BL vs CE**	***P*-value BL vs CE**	**AUC**
**0.1**	–	−4.95	0.442	+7.32	0.349	5.0
**0.2**	–	−4.95	0.230	+7.50	0.101	2.8
**0.4**	3.00	−9.8	0.047	−2.50	0.374	4.8

BL – base line, TE – tested eye, CE – control eye, IOP - intraocular pressure, AUC – area under curve of time versus response curve.

#### 1.1.19 RU 829

**Fig. 19:**
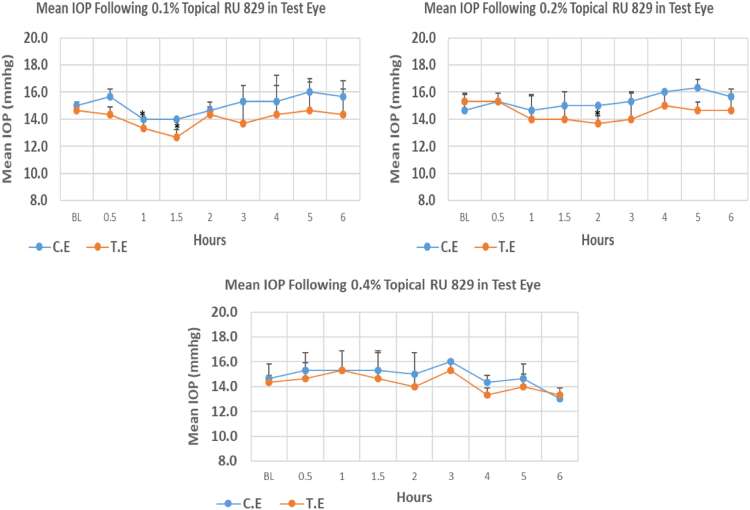
Effects of RU 829 on intraocular pressure of ocular normotensive rats in three different concentrations. *P-value≤0.05, **P-value≤0.01, and ***P-value≤0.001. TE – tested eye, CE – control eye, IOP – intraocular pressure.

RU 829 is a N9-imidazobenzimidazole derivative with molecular weight 341.2. Its 0.4% concentrations did not cause significant IOP reduction compared to baseline at any time point. Whereas, at 0.1% and 0.2% concentrations, onset of significant IOP reduction was observed at 1.00 and 2.00 h post-instillation, respectively. The 0.1% concentration of RU 829 caused higher reduction (13.63%) in IOP when compared to 0.2% and 0.4% concentrations with 10.83% and 6.98% reduction, respectively at the time of peak IOP reduction. Overall IOP lowering activities were represented by AUC values. Both 0.1 and 0.2% concentrations demonstrated higher AUC when compared to 0.4% concentration (Fig. 19, Table 19).

**Table 19:** Effects of RU 829 on intraocular pressure of ocular normotensive rats.

**RU 829 concentration %**	**Onset (h)**	**Max.** x̅ **%IOP Reduction BL vs TE**	***P*-value BL vs TE**	**Max.** x̅ **%IOP Reduction BL vs CE**	***P*-value BL vs CE**	**AUC**
**0.1**	1.00	−13.63	0.013	−2.22	0.374	6.5
**0.2**	2.00	−10.83	0.024	+11.36	0.089	6.2
**0.4**	–	−6.98	0.101	−11.37	0.067	3.0

BL – base line, TE – tested eye, CE – control eye, IOP - intraocular pressure, AUC – area under curve of time versus response curve.

#### 1.1.20 RU 832

**Fig. 20:**
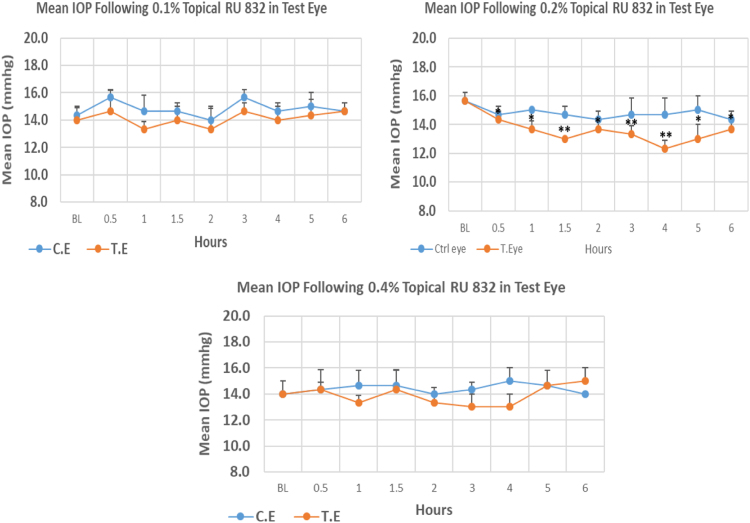
Effects of RU 832 on intraocular pressure of ocular normotensive rats in three different concentrations. *P-value≤0.05, **P-value≤0.01, and ***P-value≤0.001. TE – tested eye, CE – control eye, IOP – intraocular pressure.

RU 832 is a N9-imidazobenzimidazole derivative with molecular weight 385.3. Both 0.1% and 0.4% concentrations did not cause significant IOP reduction at any time point. The 0.2% concentration of RU 832 caused higher reduction (21.31%) in IOP when compared to 0.1% and 0.4% concentrations with 4.79% and 7.14% reduction, respectively. Overall IOP lowering activities were represented by AUC values. Both 0.1 and 0.4% concentrations demonstrated lower AUC when compared to 0.2% concentration (Fig. 20, Table 20).

**Table 20:** Effects of RU 832 on intraocular pressure of ocular normotensive rats.

**RU 832 concentration %**	**Onset (h)**	**Max.** x̅ **%IOP Reduction BL vs TE**	***P*-value BL vs TE**	**Max.** x̅ **%IOP Reduction BL vs CE**	***P*-value BL vs CE**	**AUC**
**0.1**	–	–4.79	0.374	–2.32	0.643	4.40
**0.2**	0.5	–21.31	0.002	–8.51	0.047	8.20
**0.4**	–	–7.14	0.288	+4.76	0.492	4.20

BL – base line, TE – tested eye, CE – control eye, IOP - intraocular pressure, AUC – area under curve of time versus response curve.

### 1.2 1H–Pyrimido[1,2-a]benzimidazole compounds

#### 1.2.1 RU 839

**Fig. 21:**
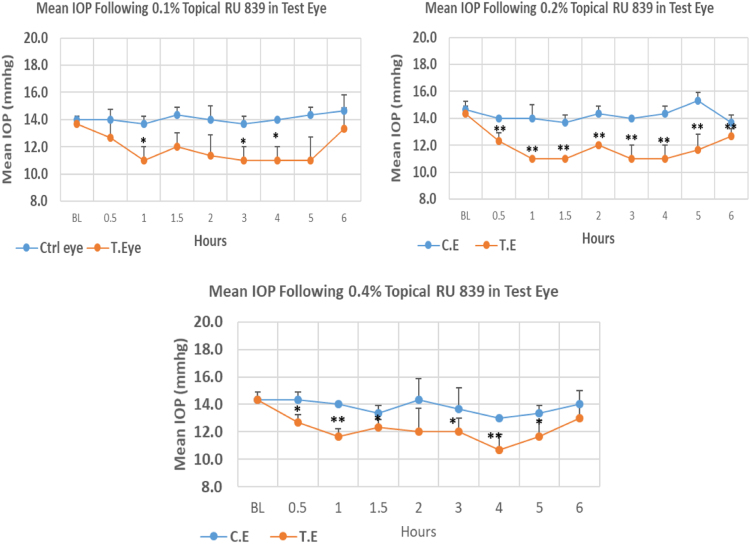
Effects of RU 839 on intraocular pressure of ocular normotensive rats in three different concentrations. *P-value≤0.05, **P-value≤0.01, and ***P-value≤0.001. TE – tested eye, CE – control eye, IOP – intraocular pressure.

RU 839 is a 1H–pyrimidobenzimidazole derivative with molecular weight 299.7. The onset of IOP lowering with 0.1%, 0.2% and 0.4% concentrations was 1.0, 0.5 and 0.5 h post-treatment, respectively. The maximum IOP reduction from baseline for 0.1%, 0.2%, and 0.4% concentrations was 19.53%, 23.24%, and 25.54%, respectively. All three concentrations showed significant differences in mean IOP values at the time of maximum reduction from baseline. Overall IOP lowering activities were represented by AUC values. Both 0.1 and 0.2% concentrations demonstrated higher AUC when compared to 0.4% concentration (Fig. 21, Table. 21).

**Table 21:** Effects of RU 839 on intraocular pressure of ocular normotensive rats.

**RU 839 concentration %**	**Onset (h)**	**Max.** x̅ **%IOP Reduction BL vs TE**	***P*-value BL vs TE**	**Max.** x̅ **%IOP Reduction BL vs CE**	***P*-value BL vs CE**	**AUC**
**0.1**	1.00	−19.53	0.016	−2.38	0.374	14.1
**0.2**	0.50	−23.24	0.001	−4.55	0.116	16.0
**0.4**	0.50	−25.54	0.008	−6.98	0.101	10.4

BL – base line, TE – tested eye, CE – control eye, IOP - intraocular pressure, AUC – area under curve of time versus response curve.

#### 1.2.2 RU 842

**Fig. 22:**
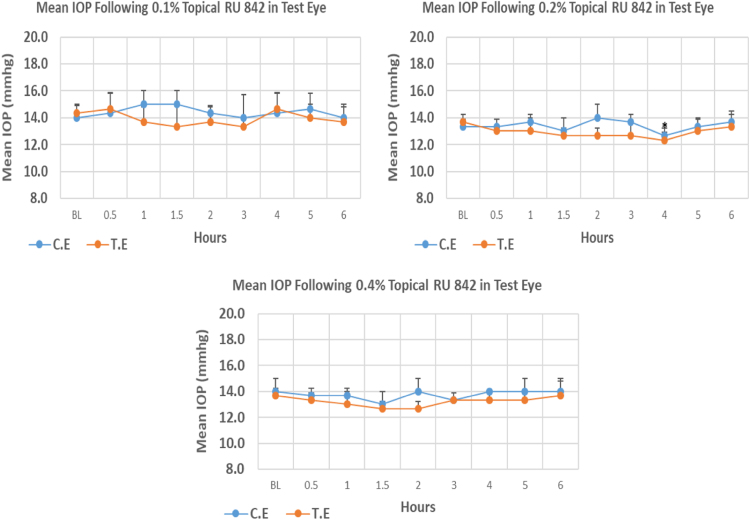
Effects of RU 842 on intraocular pressure of ocular normotensive rats in three different concentrations. TE – tested eye, CE – control eye, IOP – intraocular pressure.

RU 842 is a 1H–pyrimidobenzimidazole derivative with molecular weight 359.2. Its 0.1% and 0.4% concentrations did not cause significant IOP reduction compared to baseline at any time point. Whereas, 0.2% concentration caused onset of significant IOP reduction at 4.00 h post-instillation. The 0.2% concentration of RU 842 caused higher peak IOP reduction (9.80%) when compared to 0.1% and 0.4% concentrations with 6.98% and 7.32% reduction, respectively. Overall IOP lowering activities were represented by AUC values. An increase in AUC value was observed with increasing concentration (Fig. 22, Table 22).

**Table 22:** Effects of RU 842 on intraocular pressure of ocular normotensive rats.

**RU 842 concentration %**	**Onset (h)**	**Max.** x̅ **%IOP Reduction BL vs TE**	***P*-value BL vs TE**	**Max.** x̅ **%IOP Reduction BL vs CE**	***P*-value BL vs CE**	**AUC**
**0.1**	–	−6.98	0.349	+7.14	0.288	2.90
**0.2**	4.00	−9.80	0.047	−5.00	0.230	3.30
**0.4**	–	−7.32	0.101	−4.76	0.374	3.30

BL – base line, TE – tested eye, CE – control eye, IOP - intraocular pressure, AUC – area under curve of time versus response curve.

### 1.3 10H–Pyrimido[1,2-a]benzimidazole compounds

#### 1.3.1 RU 551

**Fig. 23:**
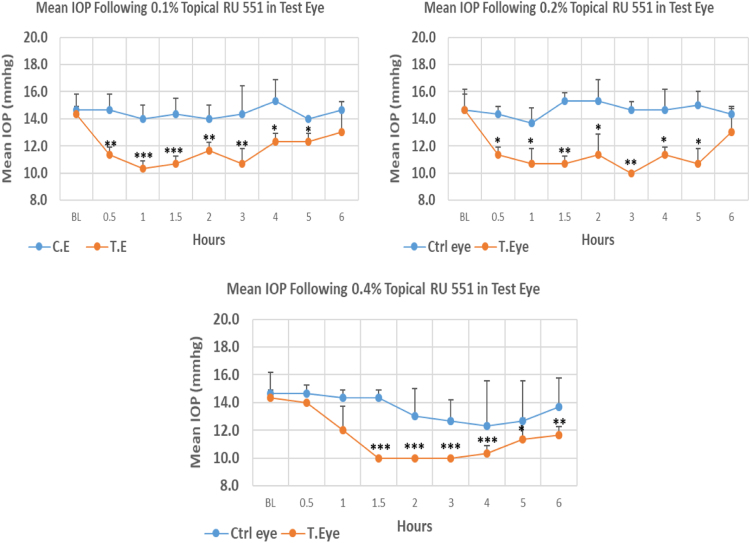
Effects of RU 551 on intraocular pressure of ocular normotensive rats in three different concentrations. *P-value≤0.05, **P-value≤0.01, and ***P-value≤0.001. TE – tested eye, CE – control eye, IOP – intraocular pressure.

RU 551 is a 10H–pyrimidobenzimidazole derivative with molecular weight 310.2. The onset of IOP lowering with 0.1%, 0.2% and 0.4% concentrations was 0.5, 0.5 and 1.5 h post-treatment, respectively. The maximum IOP reduction from baseline for 0.1%, 0.2%, and 0.4% concentrations was 27.91%, 31.83%, and 30.22%, respectively. All three concentrations showed significant differences at the time of maximum IOP reduction from baseline. Overall IOP lowering activities were represented by AUC values. Both 0.1 and 0.2% concentrations demonstrated higher AUC when compared to 0.4% concentration (Fig. 23, Table 23).

**Table 23:** Effects of RU 551 on intraocular pressure of ocular normotensive rats.

**RU 551 concentration %**	**Onset (h)**	**Max.** x̅ **%IOP Reduction BL vs TE**	***P*-value BL vs TE**	**Max.** x̅ **%IOP Reduction BL vs CE**	***P*-value BL vs CE**	**AUC**
**0.1**	0.50	−27.91	0.001	−4.55	0.492	16.30
**0.2**	0.50	−31.83	0.002	−6.82	0.417	20.30
**0.4**	1.50	−30.22	0.0002	−15.91	0.320	13.00

BL – base line, TE – tested eye, CE – control eye, IOP - intraocular pressure, AUC – area under curve of time versus response curve.

#### 1.3.2 RU 554

**Fig. 24:**
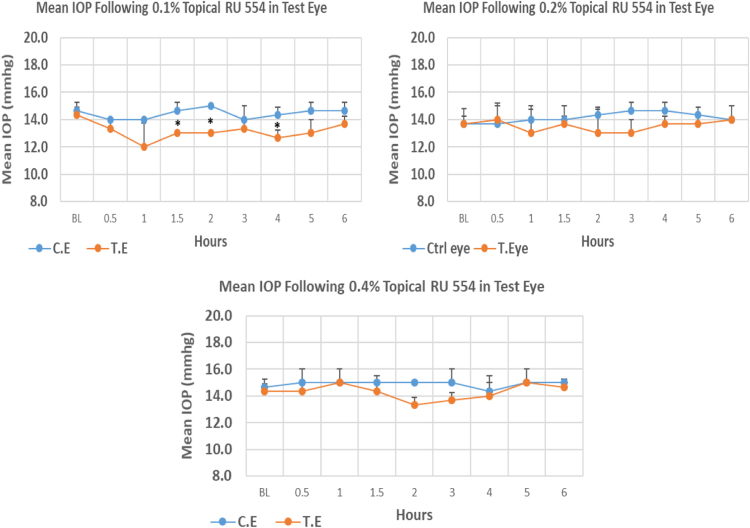
Effects of RU 554 on intraocular pressure of ocular normotensive rats in three different concentrations. *P-value≤0.05, **P-value≤0.01, and ***P-value≤0.001. TE – tested eye, CE – control eye, IOP – intraocular pressure.

RU 554 is a 10H–pyrimidobenzimidazole derivative with molecular weight 446.2. The onset of IOP lowering with 0.1%, 0.2% and 0.4% concentrations was 1.5, 1.0 and 2.0 h post-treatment, respectively. The maximum IOP reduction from baseline for, 0.1%, 0.2%, and 0.4% concentrations was 16.26%, 4.9%, and 6.98%, respectively. All three concentrations showed no significant differences in mean IOP values from baseline at the time of maximum IOP reduction. Overall IOP lowering activities were represented by AUC values. Increasing concentrations resulted in decreasing AUC value (Fig. 24, Table 24).

**Table 24:** Effects of RU 554 on intraocular pressure of ocular normotensive rats.

**RU 554 concentration %**	**Onset (h)**	**Max.** x̅ **%IOP Reduction BL vs TE**	***P*-value BL vs TE**	**Max.** x̅ **%IOP Reduction BL vs CE**	***P*-value BL vs CE**	**AUC**
**0.1**	1.5	−16.26	0.091	−4.55	0.116	8.30
**0.2**	1.0	−4.9	0.609	+2.44	0.643	4.80
**0.4**	2.0	−6.98	0.101	−2.28	0.678	3.80

BL – base line, TE – tested eye, CE – control eye, IOP - intraocular pressure, AUC – area under curve of time versus response curve.

#### 1.3.3 RU 555

**Fig. 25:**
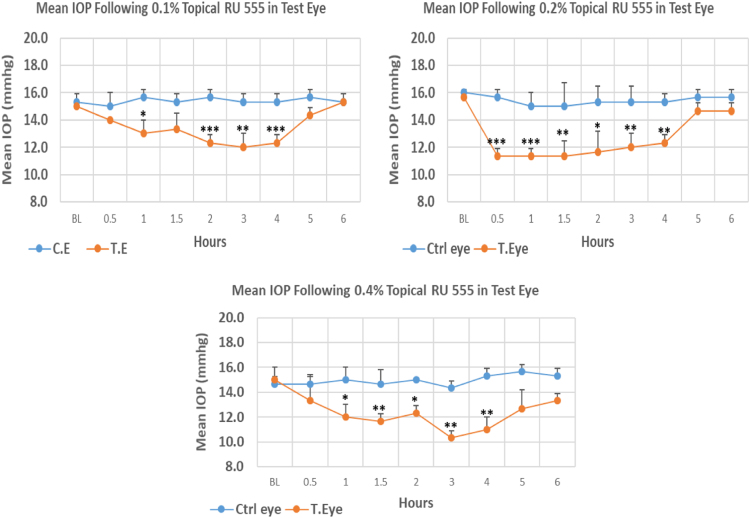
Effects of RU 555 on intraocular pressure of ocular normotensive rats in three different concentrations. *P-value≤0.05, **P-value≤0.01, and ***P-value≤0.001. TE – tested eye, CE – control eye, IOP – intraocular pressure.

RU 555 is a 10H–pyrimidobenzimidazole derivative with molecular weight 359.2. The onset of IOP lowering with 0.1%, 0.2% and 0.4% concentrations was 1.0, 0.5 and 1.0 h post-treatment, respectively. The maximum IOP reduction from baseline for 0.1%, 0.2%, and 0.4% concentrations was 20.00%, 27.70%, and 31.13% respectively. All three concentrations showed significant differences in mean IOP values from baseline at the time of maximum IOP reduction. Overall IOP lowering activities were represented by AUC of time versus concentration curve. An increase in AUC value was observed with increasing concentration (Fig. 25, Table 25).

**Table 25:** Effects of RU 555 on intraocular pressure of ocular normotensive rats.

**RU 555 concentration %**	**Onset (h)**	**Max.** x̅ **%IOP Reduction BL vs TE**	***P*-value BL vs TE**	**Max.** x̅ **%IOP Reduction BL vs CE**	***P*-value BL vs CE**	**AUC**
**0.1**	1.0	−20.00	0.007	−2.17	0.643	13.10
**0.2**	0.5	−27.70	0.001	−6.25	0.158	16.50
**0.4**	1.0	−31.13	0.002	−2.28	0.519	17.90

BL – base line, TE – tested eye, CE – control eye, IOP - intraocular pressure, AUC – area under curve of time versus response curve.

#### 1.3.4 RU 850

**Fig. 26:**
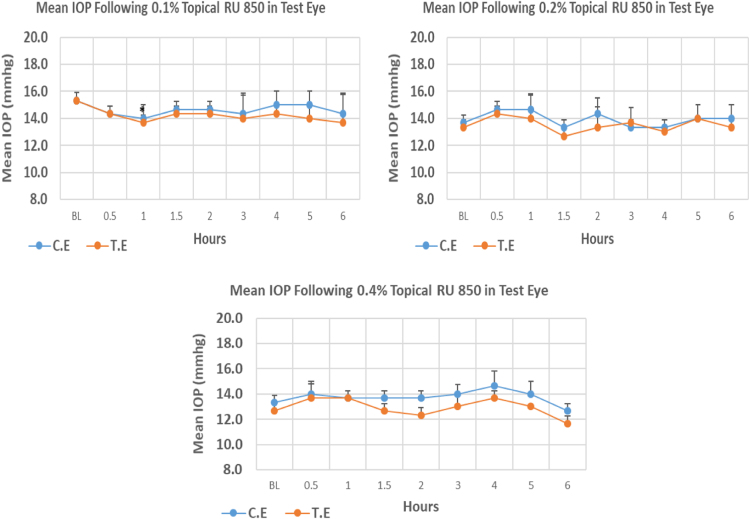
Effects of RU 850 on intraocular pressure of ocular normotensive rats in three different concentrations. *P-value ≤ 0.05, **P-value ≤ 0.01, and ***P-value ≤ 0.001. TE – tested eye, CE – control eye, IOP – intraocular pressure.

RU 850 is a 10H–pyrimidobenzimidazole derivative. At 0.2% and 0.4% concentrations this compound did not cause significant IOP reduction when compared to baseline at any time point. The 0.1% concentration of RU 850 caused higher reduction (10.83%) in IOP when compared to 0.2% and 0.4% concentrations with 4.95% and 7.89% reduction, respectively, post-instillation. Overall IOP lowering activities were represented by AUC values. An increase in AUC value was observed with increasing concentrations (Fig. 26, Table 26).

**Table 26:** Effects of RU 850 on intraocular pressure of ocular normotensive rats.

**RU 850 concentration %**	**Onset (h)**	**Max.** x̅ **%IOP Reduction BL vs TE**	***P*-value BL vs TE**	**Max.** x̅ **%IOP Reduction BL vs CE**	***P*-value BL vs CE**	**AUC**
**0.1**	1.0	−10.83	0.024	−8.69	0.116	2.90
**0.2**	–	−4.95	0.230	−2.44	0.519	2.00
**0.4**	–	−7.89	0.519	−5.00	0.230	5.30

BL – base line, TE – tested eye, CE – control eye, IOP - intraocular pressure, AUC – area under curve of time versus response curve.

### 1.4 1H–Imidazo[1,2-a]benzimidazole compound

#### 1.4.1 RU 576

**Fig. 27:**
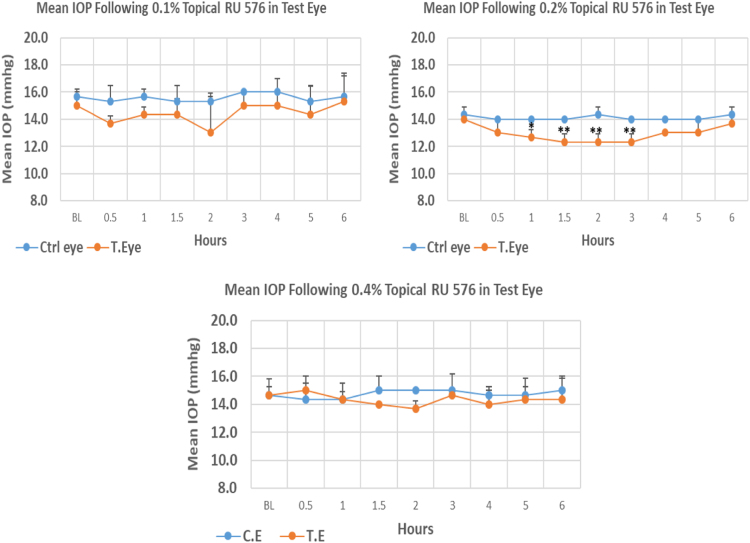
Effects of RU 576 on intraocular pressure of ocular normotensive rats in three different concentrations. *P-value≤0.05, **P-value≤0.01, and ***P-value≤0.001. TE – tested eye, CE – control eye, IOP – intraocular pressure.

RU 576 is a N1-imidazobenzimidazoles derivative with molecular weight 485.4. The onset of IOP lowering with 0.2% concentrations was 1.0 h post-treatment. Whereas at 0.1%, and 0.4% concentrations, no significant IOP reduction from baseline. The 0.1% concentration of RU 576 caused higher reduction (13.33%) in IOP when compared to 0.2% and 0.4% concentrations with 11.91% and 6.82% reduction, respectively. However only 0.2% concentration showed significant differences in maximum IOP reduction from baseline. Overall IOP lowering activities were represented by AUC values. Both 0.1 and 0.2% concentrations demonstrated higher AUC when compared to 0.4% concentration (Fig. 27, Table 27).

**Table 27:** Effects of RU 576 on intraocular pressure of ocular normotensive rats.

**RU 576 concentration %**	**Onset (h)**	**Max.** x̅ **%IOP Reduction BL vs TE**	***P*-value BL vs TE**	**Max.** x̅ **%IOP Reduction BL vs CE**	***P*-value BL vs CE**	**AUC**
**0.1**	–	−13.33	0.288	−2.13	0.519	7.10
**0.2**	1.0	−11.91	0.007	−2.32	0.374	7.60
**0.4**	–	−6.82	0.251	−2.28	0.678	2.80

BL – base line, TE – tested eye, CE – control eye, IOP - intraocular pressure, AUC – area under curve of time versus response curve.

## 2 Experimental design, materials, and methods

A total of 27 new compounds were synthesized as described previously [3], [4], [5], [6], [7], [8], [9], [10], [11] and tested for IOP lowering effect in ocular normotensive rats. These compounds included twenty 9H-imidazo[1,2-a]benzimidazoles, four 10H-pyrimido[1,2-a]benzimidazoles, two 1H-pyrimido[1,2-a]benzimidazoles and one 1H-imidazo[1,2-a]benzimidazole. All tested compounds were instilled topically at the volume of 0.5 μL.

The animal studies were done in compliance with the ARVO statement for use of animals for ocular research [17] and the institutional ethical guidelines (approval by Committee on Animal Research and Ethics (UiTM CARE 128/2015) on 11/01/2016). To evaluate 27 imidazobenzimidazoles derivatives for their IOP lowering effect, 3 different concentrations 0.1%, 0.2% and 0.4% were prepared for topical application. Among 27 compounds, 25 were water soluble and these water-soluble compounds were dissolved in 0.25% hydroxypropylmethyl cellulose (HPMC) in distilled water and the solution was filtered using 0.22 μ Millipore filter. HPMC was prepared by measuring 25 mg of HPMC and dissolving in 10 ml of distilled water. To prepare 0.4% concentration, water soluble compounds were weighed to 0.4 mg and dissolved in 1 ml (0.25%) HPMC, then serial dilution was done to obtain 0.2% and 0.1% concentrations. The remaining water insoluble compound was dissolved in 0.1% DMSO in 0.25% HPMC and similarly 3 concentrations of this compound were prepared for topical application.

**Fig. 28:**
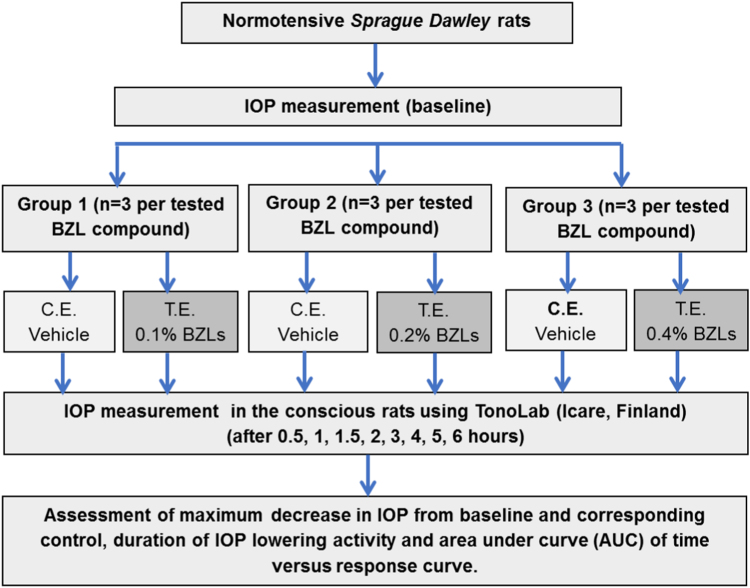
Study design to investigate the effects of imidazo[1,2-a]benzimidazole and pyrimido[1,2-a]benzimidazole compounds on intraocular pressure in ocular normotensive rats. CE – control eye, TE – tested eye, BZLs – tested benzimidazole compounds.

IOP was measured in the conscious rats using TonoLab (Icare, Finland) rebound tonometer specifically designed for rodents (rat/mouse). Since it is a noncontact tonometer, it doesn’t require use of an anaesthetic agent. The TonoLab was placed right at the centre of the cornea and the distance from the tip of the probe to surface of the cornea was 1–4 mm. For this study, 3 rats per group were used where the left eyes (TE) served as treatment eye and right eye served as control eye (CE). At each time point six readings (0.5, 1, 1.5, 2, 3, 4, 5 and 6 h after treatment with tested compound) were obtained for each eye and the mean was taken as the final measurement. The IOP lowering activities of test compounds were determined by assessing maximum decrease in IOP from baseline and corresponding control, duration of IOP lowering and area under curve (AUC) of time versus response curve (Fig. 28).
