# Supplementary material for: Data on the effects of imidazo[1,2-a]benzimidazole and pyrimido[1,2-a]benzimidazole compounds on intraocular pressure of ocular normotensive rats
Source: Data Brief. 2018 Mar 8;18:523–54. doi: 10.1016/j.dib.2018.03.019 (PMC5996230; doi:10.1016/j.dib.2018.03.019)
Supplement: Supplementary file 1 — Supplementary material. [file mmc1.docx]

**Conflict of interest**

The authors state no conflicts of interest. We reported a part of the data in the 13th Meeting of the Asia Pacific Federation of Pharmacologists (Bangkok, Thailand, 2016), the 30th and 31st Scientific Meetings of Malaysian Pharmacology and Physiology (Putrajaya, Malaysia, 2016 and Kelantan, Malaysia, 2017), the 49th Annual Scientific Congress of the Royal Australian and New Zealand College of Ophthalmologists (Perth, Australia, 2017) and International Conference on Medical and Health Sciences (Sungai Buloh, Selangor, Malaysia, 2017).
